# Long-term risk of offspring type 1 and type 2 diabetes following maternal gestational diabetes mellitus: a nationwide birth cohort study with 10-year follow-up

**DOI:** 10.1186/s12916-026-04746-7

**Published:** 2026-03-06

**Authors:** Joon Ho Moon, Han Na Jung, Bongseong Kim, Jaehyun Kim, Young Mi Jung, Hyeon Ji Kim, Jee Yoon Park, Tae Jung Oh, Soo Heon Kwak, Kyung-Do Han, Sung Hee Choi

**Affiliations:** 1 Division of Endocrinology and Metabolism, Department of Internal Medicine, Seoul National University Bundang Hospital, Seoul National University College of Medicine, 82, Gumi-Ro, 173 Beon-Gil, Bundang-Gu, Seongnam, Gyeonggi-Do 13620 Korea; 2 Department of Internal Medicine, Hallym University Sacred Heart Hospital, Hallym University College of Medicine, Anyang, Gyeonggi-Do Korea; 3 Department of Statistics and Actuarial Science, College of Natural Sciences, Soongsil University, 369 Sangdo-Ro, Dongjak-Gu, Seoul, 06978 Korea; 4 Department of Pediatrics, Seoul National University Bundang Hospital, Seoul National University College of Medicine, Seongnam, Gyeonggi-Do Korea; 5 Department of Obstetrics and Gynecology, Seoul National University Bundang Hospital, Seoul National University College of Medicine, Seongnam, Gyeonggi-Do Korea; 6 Division of Endocrinology and Metabolism, Department of Internal Medicine, Seoul National University Hospital, Seoul National University College of Medicine, Seoul, Korea

**Keywords:** Gestational diabetes mellitus, Offspring, Type 1 diabetes, Type 2 diabetes, Intergenerational

## Abstract

**Background:**

Evidence on the individual risks of type 1 and type 2 diabetes following gestational diabetes mellitus (GDM) exposure, particularly in large Asian populations, remains limited. We examined whether maternal GDM increases the risk of type 1 and type 2 diabetes in offspring in a Korean nationwide cohort.

**Methods:**

After excluding mothers with preexisting diabetes, we analyzed a nationwide Korean birth cohort of 3,491,680 mother–child pairs from 2009 to 2018, followed for up to 14 years. Type 1 diabetes was defined as International Classification of Diseases, 10th Revision (ICD-10) E10 with insulin prescription; type 2 diabetes as ICD-10 E11–E14 with antidiabetic medication use. Cox proportional hazards models were used to estimate hazard ratios (HRs) across three groups, offspring born to mothers without GDM (reference), with GDM but not treated with insulin, and with GDM treated with insulin, adjusting for maternal and neonatal covariates.

**Results:**

Of the total cohort, 424,185 (12.1%) pregnancies were complicated by GDM, among which 30,003 (7.1%) required insulin therapy during pregnancy. GDM without insulin therapy was not associated with type 1 diabetes in offspring (HR, 0.857 [95% confidence interval, 0.696–1.054]). However, offspring of GDM mothers requiring insulin during pregnancy had a higher risk of type 1 diabetes (HR, 1.936 [1.228–3.052]). For offspring type 2 diabetes, GDM without insulin during pregnancy was significantly associated with increased risk (HR, 1.281 [1.146–1.433]), with a greater risk among insulin-treated GDM pregnancies (HR, 4.329 [3.555–5.270]).

**Conclusions:**

Maternal GDM without insulin therapy is associated with an increased risk of type 2 diabetes, but not type 1 diabetes, in offspring, whereas insulin treatment for GDM during pregnancy is associated with increased risk for both type 1 and type 2 diabetes. These findings underscore the need for individualized postnatal monitoring for offspring of mothers with GDM, with heightened attention for those whose mothers required insulin therapy during pregnancy.

**Supplementary Information:**

The online version contains supplementary material available at 10.1186/s12916-026-04746-7.

## Background

Gestational diabetes mellitus (GDM) is a common pregnancy complication with its prevalence rising globally, affecting more than 10% of pregnancies. GDM is associated with adverse pregnancy outcomes, including premature delivery, primary cesarean delivery, and preeclampsia [1]. While short-term perinatal outcomes have long been studied, the long-term effects of in utero exposure to maternal hyperglycemia, particularly on offspring cardiometabolic health remain under debate [1–3].

Maternal hyperglycemia during pregnancy has been studied in association with offspring metabolic dysregulation. Early evidence from the Pima indian [4] and more recent findings from the HAPO Follow-Up Study [5] have shown that in utero exposure to GDM leads to higher glucose levels and impaired β-cell function in offspring during adolescence. The SEARCH study found that nearly half of childhood type 2 diabetes cases could be attributed to maternal diabetes and obesity during pregnancy [6]. Similarly, Canadian cohort study involving 467,850 offspring demonstrated gradual increase in the risk as well as accelerated onset of offspring type 2 diabetes born from GDM or type 2 diabetes mothers [7]. Our recent studies in a Korean population demonstrated that maternal GDM and distinct maternal metabolic phenotypes were associated with increased risks of adverse pregnancy outcomes and childhood obesity, further supporting the concept of intergenerational transmission of metabolic risk [8, 9].

Although maternal diabetes during pregnancy has been linked to offspring β-cell dysfunction and insulin resistance, most previous studies—aside from those by Wicklow and Scholtens [5, 7]—have been constrained by small sample sizes and retrospective designs. In particular, evidence distinguishing the risks of type 1 versus type 2 diabetes in offspring remains limited, especially in large-scale prospective cohorts. Furthermore, data from Asian populations are notably limited, even as the region experiences rising rates of maternal GDM and youth-onset diabetes, often occurring at relatively lower BMI thresholds compared to Western populations. To address these limitations, we conducted a Korean nationwide cohort study to investigate the association between maternal GDM status and the long-term risk of diabetes in offspring, using standardized operational definitions based on diagnostic codes and prescription records.

## Methods

### Study population

The data for this retrospective nationwide population-based cohort study were obtained from the Korea National Health Insurance Service (NHIS), which provides mandatory insurance services with a subscription rate of approximately 97% of the Korean population, covering more than 50 million individuals [10–12]. The NHIS claims database was established on January 1, 2002 and has provided nationwide population coverage since that time. It contains comprehensive sociodemographic information, healthcare utilization records including inpatient and outpatient visits, medical and surgical histories, prescription data, and diagnoses coded according to the International Classification of Diseases, 10th Revision (ICD-10). This nationally operated system enables population-wide coverage with minimal loss to follow-up over long-term observation periods. This study included births between 2009 and 2018 and the initial dataset included 3,527,248 neonates and 2,543,158 mothers. We excluded 11,770 neonates and 6,047 mothers due to missing birth dates. Additionally, we excluded 23,798 neonates and 16,296 mothers in whom there was evidence of maternal pregestational diabetes. The final analytic cohort consisted of 3,491,680 neonates and 2,520,815 mothers. The cohort was followed for up to 14 years, until the end of 2022.

### Ethics

This study was performed in accordance with the STROBE statement. All data used in this study were approved and provided in a de-identified form by the National Health Insurance Service (NHIS-2020–1–093). Approval for the present study protocol was obtained from the Institutional Review Board of Seoul National University Bundang Hospital (X-2406–906-902). The need for informed consent was waived because the NHIS database strictly adhered to the anonymization protocols outlined in the Personal Data Protection Act guidelines.

### Definition of GDM

Women with GDM were identified as follows: (1) delivered a baby between 2009 and 2018; (2) did not have a claim for diabetes mellitus based on ICD-10 codes (E10–E14) or oral antidiabetic drug or insulin use between January 1, 2002, and the index pregnancy; and (3) visited the outpatient clinic more than two times with GDM codes [13]. During the study period, GDM in Korea was predominantly diagnosed using the two-step approach with a 100-g oral glucose tolerance test in the second trimester.

### Definition of offspring type 1 and type 2 diabetes

Type 1 diabetes was defined as a diagnosis of ICD-10 E10 with concurrent insulin treatment [14, 15]. Type 2 diabetes was defined as a diagnosis of ICD-10 E11–E14 with prescription of antidiabetic medications.

### Definition of covariates

Income status was determined based on health insurance premium levels and categorized into three groups: the lowest 20% of income, recipients of public medical aid, and all other beneficiaries. Primiparity was identified based on delivery-related procedure codes recorded at the time of childbirth. Multifetal pregnancy was defined as the presence of two or more distinct neonates sharing the same mother and date of birth. Prepregnancy hypertension was defined as a diagnosis of codes I10–I13 or I15 in combination with antihypertensive medication use within one year prior to pregnancy. Dyslipidemia was defined as a diagnosis of code E78 with lipid-lowering medication use within one year prior to pregnancy. End-stage renal disease was identified based on the presence of a registration for special reimbursement codes for end-stage renal disease within one year prior to pregnancy. Depression was defined as a diagnosis of codes F32 or F33 within one year prior to pregnancy. Maternal disability was defined according to registration in the national disability registry, which requires formal assessment and certification by medical specialists [16].

### Statistical analysis

We defined three prenatal exposure groups of interest: (1) normoglycemic pregnancies without GDM, (2) GDM without insulin treatment during pregnancy, and (3) GDM requiring insulin treatment during pregnancy. These comparison groups were analyzed in multivariable models to examine their independent associations with diabetes outcomes in offspring, using normoglycemic pregnancies as the reference group. We calculated incidence rates (IR) per 1,000 person-years and applied Cox proportional hazards models to estimate hazard ratios (HRs) with 95% confidence intervals (CIs). Models were adjusted for maternal age, income, place of living, disability, depression, neonatal sex, primiparity, and pregestational hypertension and dyslipidemia. In a sensitivity analysis, offspring type 2 diabetes was alternatively defined using a more conservative operational definition based on ICD-10 code E11 in combination with prescriptions for antidiabetic medications. To further evaluate the associations of maternal and perinatal factors with the risk of offspring type 1 and type 2 diabetes, we additionally conducted covariate-specific analyses using univariate and multivariable Cox proportional hazards models. All statistical analyses were performed using SAS version 9.4 (SAS Institute, Cary, NC, USA).

### Availability of data and materials

The dataset supporting the conclusions of this article is available in the NHIS of Korea repository, [https://nhiss.nhis.or.kr/bd/ay/bdaya001iv.do], but are not publicly available due to legal and ethical restrictions under the Korean Personal Data Protection Act and NHIS data governance policies. Access is subject to review and approval by the NHIS Data Access Committee upon submission of a research proposal and institutional review board approval, and is granted under a data use agreement. Data are accessible only through secure analysis systems and cannot be downloaded or redistributed.

## Results

### Clinical characteristics of study population

A total of 3,491,680 neonates were followed for up to 14 years, with a median follow-up of 9.5 years (interquartile range, 7.0–11.8). Of these, 424,185 (12.1%) were born to mothers diagnosed with GDM, among whom 30,003 (7.1%) required insulin treatment during pregnancy. Compared with mothers without GDM, mothers with GDM, particularly those requiring insulin, were older and more likely to have multifetal pregnancy, male offspring, and higher prevalences of maternal disability and prepregnancy comorbidities such as hypertension, dyslipidemia, and depression (Table 1).

**Table 1:** Demographic and clinical characteristics according to maternal gestational diabetes or insulin treatment during pregnancy

	Total	No GDM	GDM without insulin	GDM with insulin
n	3491680	3067495	394182	30003
Maternal age	31.82 ± 4.20	31.62 ± 4.19	33.26 ± 3.89	34.20 ± 4.05
< 20	9230 (0.26)	8942 (0.29)	277 (0.07)	11 (0.04)
20–24	155483 (4.45)	147831 (4.82)	7326 (1.86)	326 (1.09)
25–29	783771 (22.45)	731880 (23.86)	48676 (12.35)	3215 (10.72)
30–34	1661730 (47.59)	1453173 (47.37)	196131 (49.76)	12426 (41.42)
35–39	764108 (21.88)	633109 (20.64)	119871 (30.41)	11128 (37.09)
≥ 40	117358 (3.36)	92560 (3.02)	21901 (5.56)	2897 (9.66)
Income				
Others	2924161 (83.75)	2563666 (83.58)	335272 (85.06)	25223 (84.07)
Lower 20%	545828 (15.63)	484294 (15.79)	56985 (14.46)	4549 (15.16)
Medical aid	21691 (0.62)	19535 (0.64)	1925 (0.49)	231 (0.77)
Geographical region				
Seoul	671015 (19.22)	585609 (19.09)	79286 (20.11)	6120 (20.40)
Metropolitan	896351 (25.67)	790843 (25.78)	98063 (24.88)	7445 (24.81)
Others	1924314 (55.11)	1691043 (55.13)	216833 (55.01)	16438 (54.79)
Primiparity	1822428 (52.19)	1602807 (52.25)	204425 (51.86)	15196 (50.65)
Multifetal pregnancy	115242 (3.30)	94955 (3.10)	18079 (4.59)	2208 (7.36)
Offspring sex (male)	1793578 (51.37)	1573637 (51.30)	203968 (51.74)	15973 (53.24)
Maternal disability	18512 (0.53)	16175 (0.53)	2135 (0.54)	202 (0.67)
Prepregnancy comorbidities				
Hypertension	17277 (0.49)	13749 (0.45)	2944 (0.75)	584 (1.95)
Dyslipidemia	9287 (0.27)	7163 (0.23)	1729 (0.44)	395 (1.32)
End-stage renal disease	226 (0.01)	197 (0.01)	28 (0.01)	1 (0.00)
Depression	56970 (1.63)	49019 (1.6)	7240 (1.84)	711 (2.37)

Variables are presented as n (%) or mean ± SD. GDM, gestational diabetes mellitus

### Offspring type 1 diabetes

The incidence of type 1 diabetes in offspring was analyzed over a total of 32,568,477 person-years of follow-up (Table 2). Among offspring born to mothers without GDM, 1166 events occurred over 29,097,043 person-years, corresponding to an IR of 0.040 per 1,000 person-years. In offspring of mothers with GDM managed without insulin, 99 events occurred over 3,216,111 person-years (IR, 0.031 per 1,000 person-years), whereas offspring of mothers with insulin-treated GDM experienced 19 events over 255,323 person-years, yielding a higher IR of 0.074 per 1,000 person-years. In multivariable-adjusted analyses, GDM without insulin treatment was not significantly associated with offspring type 1 diabetes compared with normoglycemic pregnancies (adjusted HR, 0.857; 95% CI, 0.696 to 1.054). In contrast, offspring born to mothers with insulin-treated GDM had a significantly increased risk of type 1 diabetes (adjusted HR, 1.936; 95% CI, 1.228 to 3.052).

**Table 2:** Risk of offspring diabetes according to maternal gestational diabetes and insulin treatment during pregnancy

	n	Event	Duration	Incidence rates (per 1,000 person-years)	Model 1	Model 2
Offspring type 1 diabetes						
No GDM	3067495	1166	29097042.58	0.04007	1 (ref.)	1 (ref.)
GDM without insulin	394182	99	3216111.10	0.03078	0.875 (0.712, 1.075)	0.857 (0.696, 1.054)
GDM with insulin	30003	19	255323.45	0.07442	2.022 (1.285, 3.181)	1.936 (1.228, 3.052)
Offspring type 2 diabetes						
No GDM	3067495	3125	29092162.35	0.10742	1 (ref.)	1 (ref.)
GDM without insulin	394182	352	3215497.79	0.10947	1.279 (1.145, 1.429)	1.281 (1.146, 1.433)
GDM with insulin	30003	104	255159.56	0.40759	4.384 (3.605, 5.330)	4.329 (3.555, 5.270)

Hazard ratios (95% confidence interval) were estimated using Cox proportional hazards models. Model 1 was unadjusted. Model 2 was adjusted for maternal age, income, geographical region, primiparity, offspring sex, maternal disability, and prepregnancy hypertension, dyslipidemia, and depression. GDM, gestational diabetes mellitus

### Offspring type 2 diabetes

The incidence of type 2 diabetes in offspring was evaluated over 32,562,820 person-years of follow-up (Table 2). Offspring of mothers without GDM experienced 3,125 cases during 29,092,162 person-years, corresponding to an IR of 0.107 per 1,000 person-years. Among offspring of mothers with GDM who did not require insulin treatment, 352 events were observed over 3,215,498 person-years (IR, 0.109 per 1,000 person-years). In contrast, offspring of GDM mothers with insulin treatment had 104 events over 255,160 person-years, with a markedly higher IR of 0.408 per 1,000 person-years. After multivariable adjustment, offspring born to mothers with GDM without insulin treatment had a significantly increased risk of type 2 diabetes compared with those born to mothers without GDM (adjusted HR, 1.281; 95% CI, 1.146 to 1.433). This association was stronger among offspring of mothers who required insulin during pregnancy, with an adjusted HR of 4.329 (95% CI, 3.555 to 5.270). In a sensitivity analysis using an alternative definition of offspring type 2 diabetes (ICD-10 code E11 only, instead of E11–E14), the association remained consistent with the main results, with adjusted HRs of 1.430 (95% CI, 1.238 to 1.651) for GDM without insulin and 6.051 (95% CI, 4.839 to 7.566) for GDM requiring insulin.

### Covariates

We further examined maternal and perinatal factors associated with the risk of type 1 and type 2 diabetes in offspring (Additional file 1: Supplementary Table 1). Socioeconomic disadvantages, including low income (adjusted HR, 1.204; 95% CI, 1.106 to 1.310) and public medical aid coverage (adjusted HR, 1.662; 95% CI, 1.212 to 2.279), were consistently linked to a higher risk of type 2 diabetes in offspring, whereas no clear association was observed between income level and type 1 diabetes. Female offspring exhibited a higher risk of type 1 diabetes compared to males (adjusted HR, 1.377; 95% CI, 1.233 to 1.538), but no significant association was found between sex and the risk of type 2 diabetes. Maternal dyslipidemia was associated with an increased risk of type 2 diabetes in offspring (adjusted HR, 1.677; 95% CI, 1.034 to 2.722), while its association with type 1 diabetes was attenuated and did not reach statistical significance after multivariable adjustment.

## Discussion

In this large-scale nationwide birth cohort study involving over 3.4 million mother–child pairs with up to 14 years of follow-up, we found that maternal GDM without insulin treatment was associated with a 28% increased risk of type 2 diabetes in offspring, a risk that rose significantly up to a 4.3-fold increase among children of GDM mothers who received insulin treatment during pregnancy. Although the overall risk of type 1 diabetes was not elevated in offspring of mothers with GDM without insulin, its risk increased in those born to insulin-treated GDM mothers. These findings highlight an intergenerational transmission of metabolic risk, suggesting that mothers who require insulin therapy during pregnancy, likely reflecting both genetic susceptibility and a more severe metabolic environment, may disproportionately transmit diabetes risk to their offspring, including a potential contribution to type 1 diabetes. From a clinical perspective, our results emphasize the importance of heightened attention to diabetes risk among offspring born to mothers with GDM, particularly when insulin treatment was required during pregnancy.

While GDM represents a significant metabolic disorder during pregnancy, it is not inherently an autoimmune condition. However, maternal GDM has been implicated as a potential risk factor for offspring type 1 diabetes, although the precise underlying mechanisms remain elusive. A systematic review by Hidayat et al. demonstrated that maternal GDM increases the risk of offspring type 1 diabetes by 1.66-fold with positive and linear association between maternal BMI and the risk of offspring type 1 diabetes (1.90-fold risk increase in maternal overweight and 1.25-fold risk increase in maternal obesity) [17]. Although our analysis did not show a significantly increased risk of type 1 diabetes in offspring born to mothers with GDM without insulin treatment compared to those without GDM, an elevated risk was observed among offspring of mothers who required insulin treatment during pregnancy. These findings suggest that potentially complementary pathways may underlie the association between maternal GDM and offspring type 1 diabetes, ranging from metabolic or genetic susceptibility to the influence of intrauterine hyperglycemia and exogenous insulin treatment.

Potential mechanisms by which intrauterine hyperglycemia affects fetal immune programming include epigenetic modifications and increased fetal β-cell stress or apoptosis which may lead to autoantigen exposure and compromised immune tolerance [18–20]. Recent epigenome-wide analyses have shown that maternal GDM is associated with altered DNA methylation patterns in both cord blood and placenta, accompanied by transcriptomic changes in metabolic and insulin-resistance pathways [21–23]. In our previous analysis of sibling pairs with discordant intrauterine exposure to GDM, we identified differential methylation signatures, including at the *HNF4A* locus, where methylation levels were inversely correlated with mRNA expression [24].

In addition to the effects of hyperglycemia, exogenous insulin therapy may exert distinct immunologic influences. In women with type 1 diabetes, insulin–anti-insulin IgG complexes can cross the placenta via the neonatal Fc receptor and alter fetal immune programming, potentially priming for type 1 diabetes [25, 26]. However, studies have demonstrated that insulin analogs do not cross or minimally cross the human placenta [27, 28]. This is particularly relevant for GDM, where most mothers do not have autoantibodies to β-cell, making antibody-mediated transfer an unlikely mechanism of intergenerational risk transfer in GDM. Nevertheless, exogenous insulin may still influence the intrauterine environment indirectly through placental pathways. Placental tissue expresses high amounts of insulin receptors and GDM pregnancies exposed to hyperinsulinemia and/or insulin treatment exhibits altered insulin signaling, cytokine expression, and nutrient transport, collectively altering the fetal immune–metabolic milieu [29]. Supporting this, a murine study demonstrated that maternal delivery of preproinsulin fused to an Fc-fragment can access the fetal compartment and modulate T-cell development, ultimately influencing postnatal autoimmune susceptibility [30]. Together, these findings suggest that even in the absence of direct insulin transfer, placenta-mediated alterations in cytokine signaling and immune regulation may represent a pathway through which maternal insulin therapy impacts fetal immune programming and future type 1 diabetes risk. However, these potential intrauterine influences warrant further mechanistic validation, and the observed associations may more plausibly reflect shared metabolic traits and genetic predisposition between mothers and offspring, given that maternal obesity and other metabolic disturbances were not directly adjusted for in our study.

Numerous studies have demonstrated that maternal GDM significantly increased the risk of type 2 diabetes in offspring [4–7, 31, 32]. This likely reflects more severe maternal metabolic dysfunction during pregnancy, which may indicate an increased susceptibility to diabetes in offspring [20, 33]. In HAPO Follow-up Study, higher maternal glycemic levels (fasting, 1-h, and 2-h glucose on oral glucose tolerance test during pregnancy) were positively associated with the glucose and HbA1c levels, and inversely associated with the insulin sensitivity and disposition index that measured in offspring at age 10–14 [5, 31, 34, 35]. Our recent studies in a Korean population also demonstrated that maternal metabolic perturbations including GDM and maternal obesity were associated with increased risks of adverse pregnancy outcomes including childhood obesity or large-for-gestational age, further supporting the intergenerational implications of maternal metabolic health [8, 9]. Further study is needed to determine whether interventions before or during pregnancy can prevent or mitigate the intergenerational transmission of diabetes risk.

In analyzing covariates contributing to offspring diabetes risk, maternal dyslipidemia emerged as significant factors associated with increased risks for type 2 diabetes. Maternal dyslipidemia reflects broader metabolic dysfunction during pregnancy, which can impair placental lipid processing and nutrient transport, leading to fetal lipid imbalances and increased susceptibility to metabolic disorders in offspring [36]. In addition, children from the lowest income quintile or those receiving public medical aid exhibited a higher prevalence of type 2 diabetes, highlighting the influence of social determinants on intergenerational diabetes risk. Caregivers in disadvantaged settings may have reduced health literacy and time to engage in consistent, health-promoting behaviors, which can negatively impact children's metabolic health [37]. Prior studies have shown that low-income families often face structural barriers to healthy nutrition and care, including limited access to fresh foods, higher reliance on energy-dense processed foods, and greater caregiving stress due to unstable employment or lack of support [38, 39]. Collectively, these findings indicate that maternal metabolic and socioeconomic factors may contribute to offspring diabetes risk alongside GDM and insulin treatment, underscoring the importance of a risk-stratified clinical approach that considers broader maternal health and social context.

The strengths of our study include its nationwide scale, comprehensive medical claims database coverage, and the ability to track diabetes outcomes using validated operational definitions with incorporating diagnostic codes and medication data [13–15, 40]. The inclusion of over 3 million births and over 14 years of follow-up allowed for precise estimates even for rare outcomes such as pediatric type 1 diabetes. However, several limitations warrant consideration. First, the observational nature of the study precludes definitive causal inference. Second, although we applied strict operational definitions, misclassification bias inherent to claims data cannot be excluded. Given that only 7% of women diagnosed with GDM received insulin therapy, it is possible that our GDM definition captured many low-risk cases, potentially underestimating the diabetes risk in offspring born from GDM mothers. In addition, because the incidence of both type 1 and type 2 diabetes in offspring increases markedly during adolescence, the limited duration of follow-up may have led to further underestimation of long-term risk. Taken together, these factors suggest that the observed associations may have underestimated the true intergenerational risk. Conversely, some cases classified as GDM may represent previously undiagnosed pre-existing diabetes, although we attempted to minimize this possibility by excluding women with documented diabetes diagnoses or antidiabetic medication use during the period from January 1, 2002, to the index pregnancy. Third, while the NHIS database offers the advantage of representing the general population in comparison with other large databases, potential residual confounding effects may exist owing to the absence of detailed information on family history, health behaviors, and maternal obesity, a major risk factor for offspring metabolic abnormalities.

## Conclusions

In conclusion, maternal GDM increases the risk of type 2 diabetes in offspring but not type 1 diabetes. In contrast, insulin-requiring GDM identifies a subgroup of pregnancies associated with markedly increased risk of type 2 diabetes as well as type 1 diabetes in the next generation. These findings highlight the need for tailored follow-up strategies in children born to mothers with severe forms of GDM and offer novel insights into the heterogeneity of intergenerational diabetes risk.

## Supplementary Information

Supplementary Material 1: Supplementary Table 1. Hazard ratios for offspring diabetes by maternal and perinatal factors.

Supplementary Material 2.

## Data Availability

The dataset supporting the conclusions of this article is available in the National Health Insurance Service (NHIS) of Korea repository, [https://nhiss.nhis.or.kr/bd/ay/bdaya001iv.do], but are not publicly available due to legal and ethical restrictions under the Korean Personal Data Protection Act and NHIS data governance policies. Access is subject to review and approval by the NHIS Data Access Committee upon submission of a research proposal and institutional review board approval, and is granted under a data use agreement. Data are accessible only through secure analysis systems and cannot be downloaded or redistributed.

## Abbreviations

GDM: Gestational diabetes mellitus
ICD-10: International Classification of Diseases, 10th Revision
HR: Hazard ratio
NHIS: National Health Insurance Service
IR: Incidence rate
CI: Confidence interval
