# Supplementary material for: Long-term risk of offspring type 1 and type 2 diabetes following maternal gestational diabetes mellitus: a nationwide birth cohort study with 10-year follow-up
Source: BMC Med. 2026 Mar 6;24:237. doi: 10.1186/s12916-026-04746-7 (PMC13077858; doi:10.1186/s12916-026-04746-7)
Supplement: Supplementary file 1 — Supplementary Material 1: Supplementary Table 1. Hazard ratios for offspring diabetes by maternal and perinatal factors. [file 12916_2026_4746_MOESM1_ESM.docx]

**Supplementary Table 1. Hazard ratios for offspring diabetes by maternal and perinatal factors**

|  | **Offspring type 1 diabetes** | | **Offspring type 2 diabetes** | |
| --- | --- | --- | --- | --- |
|  | Model 1 | Model 2 | Model 1 | Model 2 |
| **Maternal age (per 1 yr)** | 1.014 (1.001, 1.028)* | 1.012 (0.998, 1.027) | 1.007 (0.999, 1.015) | 1.002 (0.993, 1.010) |
| **Income** |  |  |  |  |
| Others | 1 (ref.) | 1 (ref.) | 1 (ref.) | 1 (ref.) |
| Lower 20% | 1.122 (0.972, 1.295) | 1.133 (0.980, 1.309) | 1.215 (1.117, 1.321)* | 1.204 (1.106, 1.310)* |
| Medical aid | 0.876 (0.417, 1.843) | 0.843 (0.399, 1.782) | 1.812 (1.326, 2.476)* | 1.662 (1.212, 2.279)* |
| **Geographical region** |  |  |  |  |
| Seoul | 1.082 (0.940, 1.245) | 1.077 (0.934, 1.241) | 0.818 (0.744, 0.900) | 0.826 (0.750, 0.910) |
| Metropolitan | 0.985 (0.863, 1.125) | 0.984 (0.861, 1.123) | 1.369 (1.273, 1.473)* | 1.381 (1.283, 1.485)* |
| Others | 1 (ref.) | 1 (ref.) | 1 (ref.) | 1 (ref.) |
| **Primiparity** | 0.930 (0.833, 1.037) | 0.944 (0.842, 1.058) | 0.946 (0.886, 1.010) | 0.955 (0.891, 1.023) |
| **Offspring sex (female)** | 1.378 (1.234, 1.538)* | 1.377 (1.233, 1.538)* | 1.046 (0.980, 1.116) | 1.046 (0.980, 1.117) |
| **Prepregnancy comorbidities** |  |  |  |  |
| Hypertension | 1.286 (0.642, 2.576) | 1.134 (0.560, 2.297) | 1.552 (1.063, 2.266)* | 1.332 (0.907, 1.958) |
| Dyslipidemia | 2.277 (1.085, 4.780)* | 2.122 (0.998, 4.511) | 2.024 (1.257, 3.259)* | 1.677 (1.034, 2.722)* |
| Depression | 0.919 (0.577, 1.463) | 0.904 (0.567, 1.440) | 1.235 (0.968, 1.576) | 1.193 (0.935, 1.523) |

Hazard ratios (95% confidence interval) were estimated using Cox proportional hazards models. Model 1 was unadjusted. Model 2 was adjusted for maternal age, income, geographical region, primiparity, offspring sex, maternal disability, and prepregnancy hypertension, dyslipidemia, and depression. GDM, gestational diabetes mellitus. **P* <0.05.
